# Effects of oxygen-glucose deprivation (OGD) on barrier properties and mRNA transcript levels of selected marker proteins in brain endothelial cells/astrocyte co-cultures

**DOI:** 10.1371/journal.pone.0221103

**Published:** 2019-08-19

**Authors:** Erica Tornabene, Hans Christian Cederberg Helms, Stine Falsig Pedersen, Birger Brodin

**Affiliations:** 1 Department of Pharmacy, Faculty of Health and Medical Sciences, University of Copenhagen, Copenhagen, Denmark; 2 Department of Biology, Faculty of Science, University of Copenhagen, Copenhagen, Denmark; Texas Tech University, UNITED STATES

## Abstract

Ischemic stroke has been shown to induce breakdown of the blood-brain barrier, although these changes are not fully characterized. Oxygen-glucose deprivation (OGD) has been used to investigate the effects of ischemia in cultured brain capillary endothelial cells, however this involves a change of medium which in itself may affect the cells. The aim of the present study was to investigate the effect of OGD and simple medium exchange followed by 48 h of reperfusion on barrier properties of primary bovine endothelial cells co-cultured with rat astrocytes. Barrier properties were evaluated by transendothelial electrical resistance measurements, passive permeability of flux markers, RT-qPCR and immunocytochemistry. Both OGD and simple medium exchange caused an increase in endothelial monolayer permeability. This correlated with reduced transcript levels of a number of tight junction and tight junction-associated proteins (claudin-1, claudin-5, occludin, ZO-1, tricellulin, marveld3 and PECAM-1), as well as with altered transcript level of several transporters and receptors (GLUT-1, HB-EGF, InsR, TfR, two members of the low density lipoprotein receptor family, LDLR and LRP-1, and the efflux transporter BCRP). In contrast, effects induced specifically by OGD were transient de-localization of claudin-5 from the junction zone, increased InsR localization at the plasma membrane and transient downregulation of MRP-1 and P-gp transcript levels. In conclusion, OGD caused changes in claudin-5 and InsR localization, as well as in MRP-1 and P-gp transcript levels. Our results however also indicated that medium exchange alone caused changes in functional barrier properties and expression levels of wide range of proteins.

## Introduction

Brain capillary endothelial cells provide a barrier between the blood and the brain parenchyma, and thus control exchange of solutes and protect the brain tissue against potentially neurotoxic compounds circulating in the blood stream. This blood-brain barrier (BBB) function of capillary endothelial cells is due to their unique characteristics including lack of fenestrations, decreased pinocytotic activity and the presence of tight junctions (TJs), efflux proteins of the ATP-binding cassette (ABC) type and metabolizing enzymes [1]. Endothelial cells at the BBB are in close contact with two other cell types, pericytes and astrocytes and, together with neurons, microglia and extracellular matrix components, constitute the neurovascular unit (NVU). Permeability properties of the capillary endothelium may be altered in pathophysiological conditions, thus compromising the neuroprotective activity of the BBB. Enhanced paracellular diffusion across the BBB has been observed during ischemic stroke, suggesting that perturbation of TJ complexes may play a role in stroke pathology [2, 3].

At present, we have a limited understanding of the pathophysiological changes in paracellular permeability and protein expression occurring in brain capillary endothelial cells during a focal ischemic stroke. It is therefore of great interest to study perturbations in BBB properties under ischemic conditions in order to elucidate the pattern of changes at a molecular level. Due to the complex pathology of ischemic stroke which makes it difficult to control experimental variables, and the experimental challenges involved in isolating native endothelial cells without other contaminating cell types, a number of *in vitro* models of the ischemic BBB have been developed (for references, see [4]).

Oxygen-glucose deprivation (OGD) treatment is the most commonly used method for inducing ischemic injury *in vitro* [5]. In practice, this treatment consists of replacement of the standard culture medium with a hypoxic N_2_/CO_2_ equilibrated culture medium without glucose, and the incubation of the cells in a hypoxic chamber having the same N_2_/CO_2_ gas combination. The majority of *in vitro* BBB models are based on endothelial cell monolayers cultured on permeable supports in 2-compartment culture systems, alone or in combination with other NVU cell types. The replacement of the culture medium is performed by aspirating up the medium and subsequently adding the new medium to the culture system. This operation might however be a source of stress for endothelial cell monolayers, since fluid turbulences can be generated during the replacement of the medium. Peripheral endothelial cells have been shown possess different molecular sensors which enable them to transduce mechanical stimuli into intracellular responses via activation of various signaling transduction pathways [6–8]. Alterations in the blood flow and consecutively, in the shear stress exerted on the endothelial luminal surface have been linked to different cardiovascular pathologies, including atherosclerosis (for references see [9]). It seems therefore reasonable to postulate that the replacement of culture medium necessary for inducing OGD and the concurrent turbulent shear stress generated might affect the endothelial cell barrier properties, thus obscuring the effects of the OGD treatment.

The interpretation of the results from studies using cultured endothelial cells treated with OGD for mimicking the ischemic BBB has furthermore been complicated by the fact that some of the employed brain endothelial cell lines have a low barrier tightness compared to the *in vivo* situation, making investigation of variations in barrier properties difficult.

Bovine endothelial monolayers, co-cultured with astrocytes, can obtain a low barrier permeability and thus also a high transendothelial resistance, with values in the range of 1000–2000 Ω∙cm^2^ [10, 11], which approaches estimated *in vivo* values of ~2000 Ω∙cm^2^ (determined in pial vessels) [12].

The aim of the present study was to investigate the changes in barrier properties as well as in the localization and transcript level of selected junction proteins, transporters and receptors induced by experimental OGD and reperfusion, and to compare them with the changes observed upon medium exchange in a high-resistance brain endothelial/astrocyte co-culture cell model.

Overall, our findings indicate that both the OGD treatment and simple medium exchange cause a transient breakdown of *in vitro* barrier function. Surprisingly, HB-EGF, a receptor that has been shown to undergo upregulation in many brain diseases including ischemia [13, 14] was upregulated both by OGD and medium exchange. Claudin-5 reacted specifically to the OGD by translocating to the cytosol during the treatment and redistributing to the junctional zones in the subsequent reperfusion phase, whereas the InsR showed increased membrane localization following OGD. Apart from these OGD-specific alterations, a large number of marker protein changes, as well as changes in barrier properties, took place both after OGD and simple medium exchange. Our results stress the importance of medium exchange controls in OGD experiments and suggest the need to reevaluate previous conclusions from experiments lacking such controls.

## Materials and methods

### Materials

NucleoSpin RNA/Protein isolation kit was from MACHEREY-NAGEL (Tilst, Denmark), RO-20-1724 was from Calbiochem (San-Diego, USA). All primers were from Invitrogen (Naerum, Denmark), High Capacity cDNA Reverse Transcription Kit was from Applied Biosystems (Naerum, Denmark). Powdered Dulbecco's Modified Eagles Medium was from Gibco (Breda, Netherlands). Filters of 160 μm pore size was obtained from Millipore (Copenhagen, Denmark), filters of 200 μm pore size from Merrem & La Porte (Zaltbommel, Netherlands). FastStart Essential DNA Green Master for RT-qPCR was purchased from Roche Diagnostics (Hvidovre, Denmark). Paraformaldehyde, 16% w/v aq. soln. was from Alsa Aesar (Karlsruhe, Germany), Triton X-100 Molecular Biology grade was from AppliChem (Herlev, Denmark). Rabbit polyclonal anti-Claudin-5: ab15106, mouse monoclonal anti-Insulin Receptor-β [C18C4]: ab69508, rabbit polyclonal anti-HB-EGF: ab92620, rabbit polyclonal anti-VWF: ab6994 were from Abcam (Cambrige, United Kingdom), rabbit polyclonal anti-Glut-1: PA1-1063 and rabbit polyclonal anti-ZO-2: PA5-17155 were from ThermoFisher (Slangerup, Denmark), mouse monoclonal anti-P-gp: NB600-1036 was from Novus Biologics (England, United Kingdom). Alexa Fluor-488 goat anti-mouse IgG (H+L): A11001 and Alexa Fluor-488 goat anti-rabbit IgG (H+L): A11008 were from Invitrogen (Naerum, Denmark). Fetal bovine serum (FBS) was from PAA-Laboratories (Pasching, Austria). [^14^C]-Mannitol and Ultima Gold Scintillation fluid were from Perkin Elmer (Hvidovre, Denmark). Collagenase type III, Trypsin TRL and DNAse I were from Worthington (Lakewood, USA). All other chemicals were from SIGMA-ALDRICH (Steinheim, Germany), unless otherwise stated.

### Cell culture

#### Isolation of bovine brain capillaries and astrocytes

Primary brain capillary endothelial cells were isolated from calf brains (<12months) and sacrificed at a slaughterhouse (Mogens Nielsen Kreaturslagteri A/S, Herlufmagle, Denmark) as previously described [15]. Briefly, after removal of meninges from the brain surface, gray matter from the cortex was scraped off with a scalpel and homogenized in a Dounce tissue grinder (Wheaton Science Products, Millville, USA). The homogenate was filtered using filters of 160 μm mesh (Millipore, Cat. NY6H00010) and the filters were flushed with DMEM-comp to collect the vessel fraction. The vessel fraction was centrifuged for 5 min at 500g (4°C) and the resultant pellet was resuspended in an enzyme mix containing DNase I (170 U∙ml^-1^), Collagenase type III (200 U∙ml^-1^) and trypsin TRL (90 U∙ml^-1^) and incubated for 1 hour at 37°C. The suspension was filtered through 200 μm mesh (Merrem & la Porte, Zaltbommel, Netherlands) and centrifuged for 5 min at 500g (4°C). Finally, the pellet containing brain capillaries was resuspendend in freezing medium (10% dimethyl sulfoxide and 90% FBS), aliquoted in vials and frozen at -80°C. The day after, the vials were transferred to liquid nitrogen where they were stored.

Astrocytes were isolated from 2–3 days old Sprague Dawley rats (Taconic, Ejby, Denmark), and cultured until confluence in T75 flasks as previously described [16]. The personnel involved in isolation of rat brain astrocytes had received training according to European guidelines (FELASA C). In the third week of astrocyte culture, after change to FBS concentration of 10%, medium was collected every second day as astrocyte-conditioned medium (ACM) and stored at -20°C. After three weeks of culture, the astrocytes were then trypsinized, resuspended in dimethyl sulfoxide:FBS (1:9), aliquoted in cryovials with approximately 1.5–2·10^6^ cells per vial and frozen overnight at -80°C. The astrocytes were then stored in liquid nitrogen until use.

#### Co-culture of brain endothelial cells and astrocytes

Primary brain capillary endothelial cells were seeded on collagen type IV/fibronectin coated T75 flask and cultured in GM+ containing 4 μg/ml of puromycin (10% CO_2_, 37°C). After 2 days, the medium was replaced with fresh medium without puromycin. Cryopreserved astrocytes were thawed and seeded on the lower surface of collagen IV/fibronectin coated Transwell inserts (surface area = 1.12 cm^2^, pore size = 0.4 μm, Corning, Schiphol, The Netherlands) at a density of 100,000 cell∙cm^-2^ and cultured in DMEM-comp medium (10% CO_2_, 37°C). When the endothelial cells had reached 60–80% confluence, they were trypsinized, seeded on the upper surface of the filter inserts at a density of 90,000 cells∙cm^-2^ insert and cultured in the co-culture system in GM-. At day 3 of co-culture, the growth medium was replaced with differentiation medium (DM-TES). For permeability studies, the medium utilized was DM-TES without phenol red. After 3 days (day 6 of co-culture), the cells were subjected to oxygen-glucose deprivation (OGD).

### Oxygen-glucose deprivation/reperfusion

OGD experiments were performed in a hypoxia workbench (X3 Xvivo System, BioSpherix, NY, USA), with an atmosphere of 1% O_2_, 10% CO_2_ and 89% N_2_ and a temperature of 37°C. The differentiation medium (DM-TES) was replaced with aglycemic medium let equilibrate in the hypoxia workbench for the OGD-treated cells. After 4 h of treatment, a concentrated solution of glucose was added to the cell medium of the OGD-treated cells in both apical and basolateral chambers, in order to restore the standard concentration of 25 mM, and the cells were transferred back to the normal incubator (90% room air-10% CO_2_, 37°C) for 24 or 48 h in order to mimic a reperfusion period. For medium exchange treatment, monolayers were subjected to medium replacement with fresh DMEM-TES (or DM-TES without phenol red in case of permeability studies) and were incubated in a standard incubator (90% room air-10% CO_2_, 37°C) for 4, 24 or 48 h.

### Transendothelial electrical resistance (TEER) measurement

The TEER was measured using an EndOhm Chamber connected to an EVOM2 Meter (World Precision Instrument, England, UK). At day 6 of co-culture, the resistance was monitored throughout the experimental procedure, in particular before the treatments (t0), after 4 h of OGD/medium exchange, and after 24 and 48 h of reperfusion.

### Permeability assay

Passive permeability (P_app_) of selected marker molecules ([^14^C]-mannitol, FITC-dextran 4, FITC-dextran 40 and FITC-dextran 150) was determined. 20x stock solution of [^14^C]-mannitol (20 μCi∙ml^-1^) and 10x stock solution of fluorescent compounds FITC-dextran 4, FITC-dextran 40 and FITC-dextran 150 (10 mg∙ml^-1^) were prepared in DM-TES without phenol red. 25 μL of [^14^C]-mannitol stock solution or 50 μL of FITC-dextran 4, FITC-dextran 40 or FITC-dextran 150 stock solution were added to the apical culture medium and the cell plate was placed in a shaking table with a speed of 90 rpm, a controlled temperature of 37°C in atmospheric air. Receiver samples of 100 μL were collected from the basolateral medium at different time points (15, 30, 45, 60, 90 and 120 min) and replaced with clean DM-TES without phenol red. At the end of the experiment (120 min), [^14^C]-mannitol donor samples of 20 μL were collected. 2 mL of ultima gold scintillation fluid (Perkin-Elmer) were added to each sample and the disintegrations counted by a Tri-Carb 2910 TR Liquid Scintillation Analyzer provided by Perkin Elmer (Waltham, MA, USA) were converted to transported amounts using the specific activity of [^14^C]-mannitol (1.22∙10^11^ DPM∙mmol^-1^). Regarding FITC-dextrans, donor samples of 5 μl were withdrawn from the apical side and diluted 1:400 at the end of the experiment. The receiver samples and the diluted donor samples were pipetted to a black, flat-transparent 96-well plate and the relative fluorescence was measured using the plate reader NOVOStar (excitation 485 nm, emission 520 nm) (RAMCON A/S Denmark).

### Transcript level analysis

The RNA was isolated using NucleoSpin RNA/Protein kit (MACHEREY-NAGEL, Tilst, Denmark) according to the manufacturer’s protocol.

The concentration and purity of the isolated RNA was evaluated at NanoDrop 2000c (ThermoFisher, Hvidovre, Denmark). Subsequently, the RNA was reverse transcripted in cDNA with the High Capacity cDNA Reverse Transcription Kit (Applied Biosystems, Naerum, Denmark). 0.5 μg RNA was used for each reaction. Real Time Quantitative PCR (RT-qPCR) reactions were carried out using DNA Green Master for RT-qPCR according to the manufacturer’s protocols (Roche Diagnostics, Hvidovre, Denmark). For this aim, primers specifically designed for bovine and validated with standard curves were employed (for primer sequences see the S2 Table).

Normalized relative quantities were determined relative to hypoxanthine phosphoribosyltransferase 1 (HPRT-1), succinate dehydrogenase complex flavoprotein subunit A (SDHA) and tyrosine 3-monooxygenase/tryptophan 5-monooxygenase (YWHAZ) reference genes as explained in the “Data treatment” section. RT-qPCR was performed using a Roche LightCycler 96 (Roche, Pleasanton, USA).

### Immunocytochemistry

Endothelial cells co-cultured with astrocytes in coated filter inserts were fixed with 4% paraformaldehyde in HBSS buffer for 15 min, permeabilized with 0.1% Triton 100x in PBS for 5 min and blocked for 30 min at room temperature with PBS supplemented with 2% bovine serum albumin (BSA). The filters were removed from the supports and cut in small pieces for antibody incubations over night at 4°C (antibody details are given in S3 Table). The samples were washed 3 times with 2% BSA in PBS and incubated 45 min at room temperature with Alexa-488 labelled secondary antibodies together with propidium iodide (1:500). The filters were rinsed three times with 2% BSA in PBS, placed on glass slides, sealed with nail polish, and then examined at confocal laser scanning microscopy (Carl Zeiss, LSM 510, Jena, Germany).

The quantification of the number of pixels present inside single cells for the analysis of claudin-5 and ZO-2 cytosolic signal was performed using the program ImageJ.

### Cell density

The cell density was extrapolated from the images obtained at confocal microscopy by manually counting the number of cells from an average of 5 images (size: 2.1∙10^−4^ cm^2^) for each condition (t0, 4h OGD/medium exchange and 24/48 h R).

### Data treatment

The TEER data were normalized by subtracting the resistance of an empty filter and multiplying for the diameter of the filter insert.

Apparent permeability (P_app_) of the selected marker molecules was calculated by using the simplified Fick’ law equation:

$$J=Papp*Cdonor=>Papp=\frac{J}{Cdonor}$$

where J represent the flux at steady-state and C_donor_ is the concentration in the donor compartment.

Steady-state fluxes were determined by plotting the accumulation of the tested compound in the receiver chamber (mol∙cm^-2^) against the time (min), and calculating the slope of the linear region. Donor samples were collected at the beginning and end of each experiment to make sure that sink conditions were applicable (less than 10% of total amount transported).

Regarding the RT-qPCR data, efficiency for all genes of interest and reference genes were calculated by the equation E = 10 ^(-1/slope)^, where the slope was found from a ten-fold serial dilution calibration curves. The normalized relative quantities where then extrapolated from the quantification cycle values (Cq) using a model previously described [17] with some few modifications [18, 19], adjusting for differences in PCR efficiency between the gene of interests and the reference genes (HPRT1, SDHA and YWHAZ).

The cell density was obtained by relating the number of cell counted in the confocal images to the cross-section area of the filter insert using the proportion:

Ncellcounted:Apicture=Ncell/filter:Afilter

$$=>Ncell/filter=\frac{Ncell\;counted*Apicture}{Afilter}$$

Finally, the quantification of the intracellular signal of claudin-5 and ZO-2 was performed using ImageJ software. The intracellular space of single cells was selected manually by drawing a line along the cell contour as close as possible to the cellular edge but not overlapping the junctional zone, as determined by visual inspection. The mean pixel intensity for the green channel in the selected area was calculated by the program. An average of 3 cells per image were analyzed.

Data from all experiments were expressed as mean ± standard error of mean (SEM) and analyzed by one-way ANOVA using GraphPad Prism Software Inc. (La Jolla, CA, USA). Multiple comparison tests were performed with Dunnett’s post-test for comparisons against t0 columns, and with Bonferroni’s post-test for comparison of each pair of columns. P values less than 0.05 were considered statistically significant. In figure texts, “N” was used for the number of repetitions, while “n” was for the number of individual experiments.

## Results

### The permeability of endothelial cell monolayers increased to a similar degree after simple medium exchange and OGD treatment

**Fig 1:**
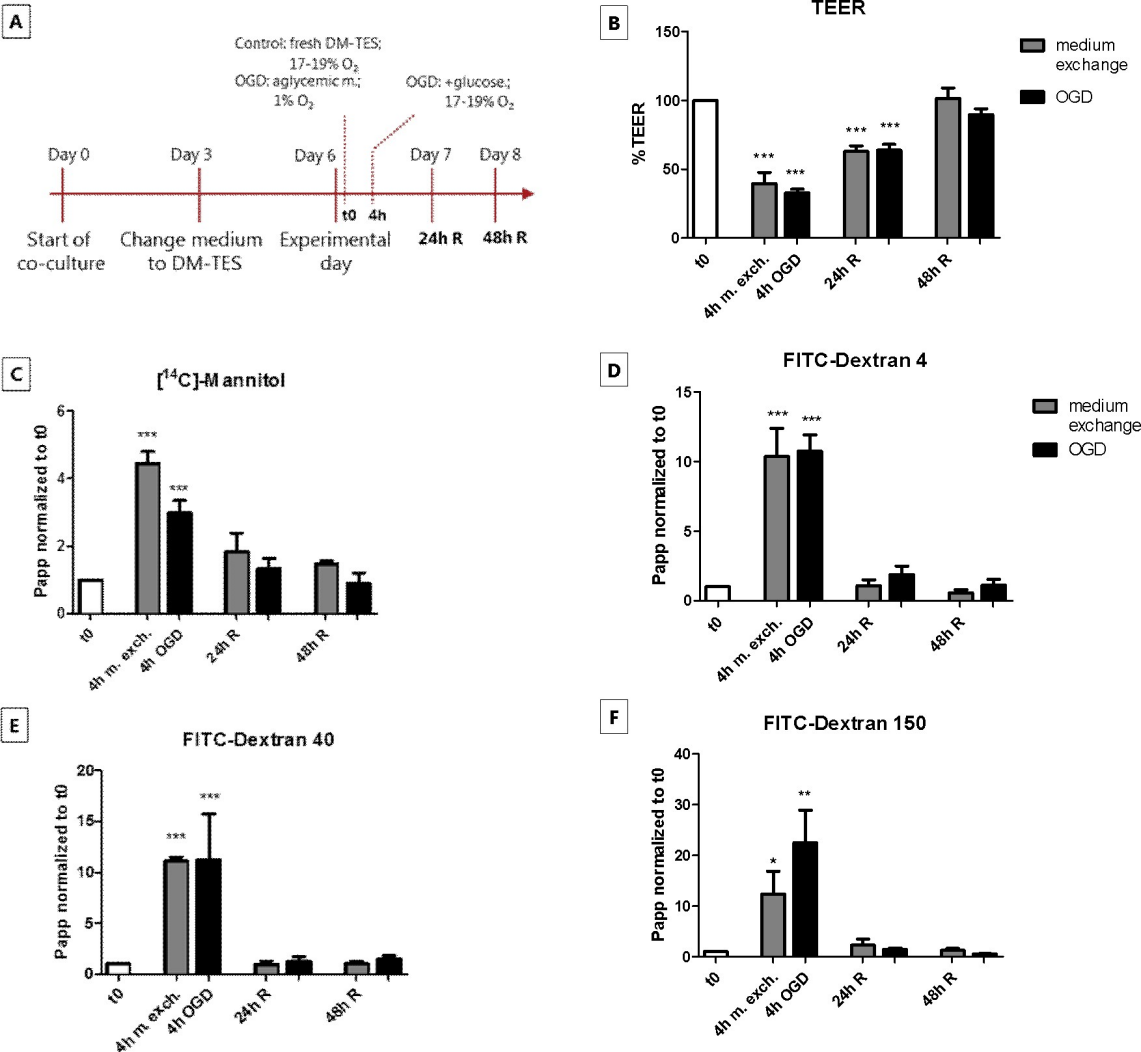
Transendothelial electrical resistance (TEER) and permeability of paracellular flux markers across brain endothelial cell monolayers in co-culture, during *in vitro* oxygen-glucose deprivation (OGD) and medium exchange treatments. (A) Schematic overview of OGD and medium exchange protocols. At day 0, the endothelial cells were seeded on filter inserts in contact co-culture with astrocytes. At day 3 of co-culture, the cell medium (GM-) was changed to DM-TES medium. After additional 3 days (day 6), when the monolayers had developed an optimal resistance, the cells were exposed to OGD or subjected to simple medium exchange as control. After 4 h, standard culture conditions were reestablished for the OGD treated cells, in order to mimic the reperfusion phase (R). The cells were checked after 24 and 48 h of reperfusion. (B) TEER and (C-F) paracellular permeability of [^14^C]-mannitol, FITC-dextran 4, FITC-dextran 40 and FITC-dextran 150 across endothelial monolayers were monitored immediately before OGD/medium exchange (t0), after 4 h of OGD/medium exchange (4h OGD/m. exch.) and after 24 h and 48 h of reperfusion (24/48 h R). Bar graphs represent means normalized to t0 and error bars are +SEM. N = 12–24, n = 11–12 (B); N = 3–4, n = 3–4 (C-F). The white bars show the values at t0, the gray bars show the cells subjected to medium exchange only, while the black bars represent the cells treated with OGD. Columns were compared to t0 using one-way ANOVA and Dunnett’s multiple comparison post-test. *: p<0.05, **: p<0.01, ***: p<0.001. Each pair of columns was compared using one-way ANOVA with Bonferroni’s post-test.

The effects of OGD and reperfusion on paracellular permeability in the BBB co-culture model were investigated and compared to the effects of aspiration and replacement of culture medium as outlined in Fig 1A.

The resistance of the co-cultured monolayers was measured before the treatments, after 4 h of OGD/medium exchange, and after 24 and 48 h of *in vitro* reperfusion (Fig 1B). The initial TEER had a mean value of 1118 ± 112 Ω∙cm^2^ (n = 14). The resistance decreased significantly after both 4 h of OGD (362 ± 43 Ω∙cm^2^) and medium exchange (349 ± 76 Ω∙cm^2^), as compared to the TEER at time zero (t0). After 24 h of reperfusion, the TEER increased to 691 ± 67 Ω∙cm^2^ in the cell treated with OGD and to 581 ± 57 Ω∙cm^2^ in the cells subjected to medium exchange, and after 48 h of reperfusion it returned to the initial levels in both experimental set up (928 ± 89 Ω∙cm^2^ in the OGD-treated cells and 929 ± 92 Ω∙cm^2^ in the cells that had undergone medium exchange). The passive permeability of the markers [^14^C]-mannitol (180 Da), FITC-dextran 4 (4 kDa), FITC-dextran 40 (40 kDa) and FITC-dextran 150 (150 kDa) was measured at different time points during the OGD and reperfusion phases (Fig 1C–1F).

The absolute permeabilities at t0 were 7.3·10^−7^ ± 1.3·10^−7^ cm^2^∙s^-1^ for [^14^C]-mannitol, 1.6·10^−7^ ± 0.4·10^−7^ cm^2^∙s^-1^ for FITC-dextran 4, 1.1·10^−7^ ± 0.3·10^−7^ cm^2^∙s^-1^ for FITC-dextran 40 and 0.4·10^−7^ ± 0.2·10^−7^ cm^2^∙s^-1^ for FITC-dextran 150. After 4 h of OGD treatment, the permeability of [^14^C]-mannitol, FITC-dextran 4, FITC-dextran 40 and FITC-dextran 150 increased with a factor of 3, 10, 11 and 21, respectively, as compared to t0. Similarly, in the monolayers subjected to medium exchange the marker permeabilities increased with a factor of 5, 10, 11 and 11, respectively. After 24 h of reperfusion, the permeability values returned to baseline levels and remained stable also after 48 h in both the OGD- and medium exchange-treated cells. It has previously been shown that medium exchange can cause a decrease in transendothelial resistance in cultured endothelial monolayers [11] as well as in other cultured cell monolayers [20]. The observed changes in TEER and permeability during and after the OGD treatment were therefore likely to be a combination of the OGD and the medium exchange.

### The transcript level of tight junction proteins changed in a similar manner after medium exchange and OGD treatment

**Fig 2:**
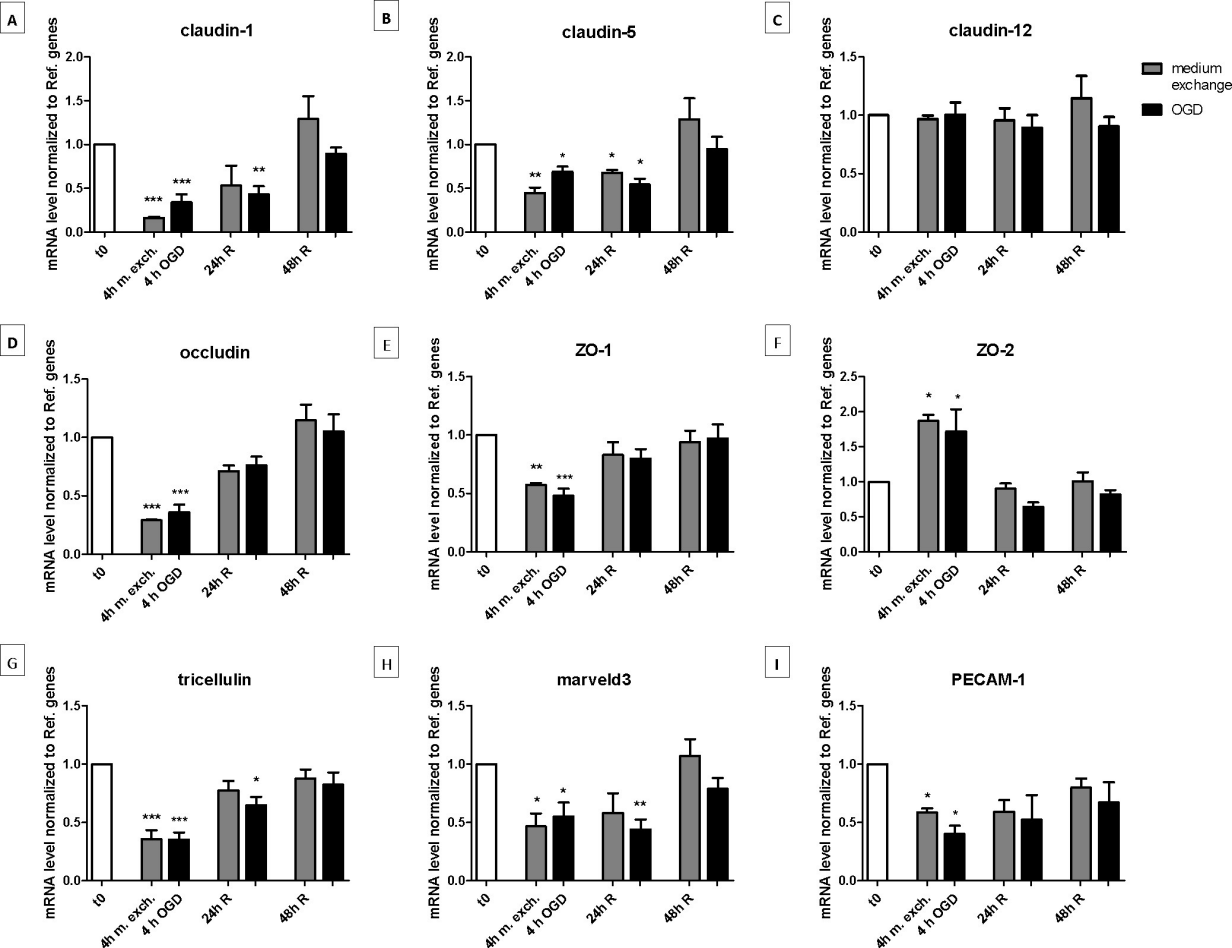
Transcript levels of selected junction proteins in endothelial cells co-cultured with astrocytes before, during and after oxygen-glucose deprivation (OGD) treatment or medium exchange. Endothelial cells which had undergone OGD (black bars) or medium exchange (gray bars) were collected for mRNA isolation. After the reverse transcription of the mRNA, the resultant cDNA was quantified by RT-qPCR using primers specifically designed for bovine. The mRNA amounts of claudin-1 (A), claudin-5 (B), claudin-12 (C), occludin (D), ZO-1 (E), ZO-2 (F), tricellulin (G), marveld3 (H) and PECAM-1 (I) were normalized to reference genes (for calculation see “Materials and methods” section). Bar graphs represent means and error bars are +SEM. The white bars show the values at t0, the gray bars show the cells subjected to medium exchange, while the black bars show the OGD treated cells. Columns were compared to t0 using one-way ANOVA and Dunnett’s multiple comparison post-test. *: p<0.05, **: p<0.01, ***: p<0.001. Each pair of columns were compared using one-way ANOVA with Bonferroni’s post-test.

The transcript level of a number of brain endothelial TJ and TJ associated proteins were investigated (Fig 2). The majority of the transcripts (claudin-1, -5, occludin, zonula occludens-1 (ZO-1), tricellulin, marveld3 and platelet endothelial cell adhesion molecule (PECAM-1) were significantly downregulated after both 4 h of OGD and medium exchange, and returned to baseline levels 48 h after reperfusion (previous Fig 1B). Conversely, zonula occludens-2 (ZO-2) transcript level increased after both treatments, and it returned to baseline levels upon reperfusion (Fig 2M). Claudin-12 remained unchanged during all phases of both OGD and medium exchange experimental set up.

Summarizing, monolayers that had undergone medium exchange displayed similar changes as those observed under OGD, in all investigated TJ related transcripts, indicating that the observed changes can be explained by the medium change on its own.

### The change in permeability after OGD/medium exchange correlated with a change in TJ protein localization

**Fig 3:**
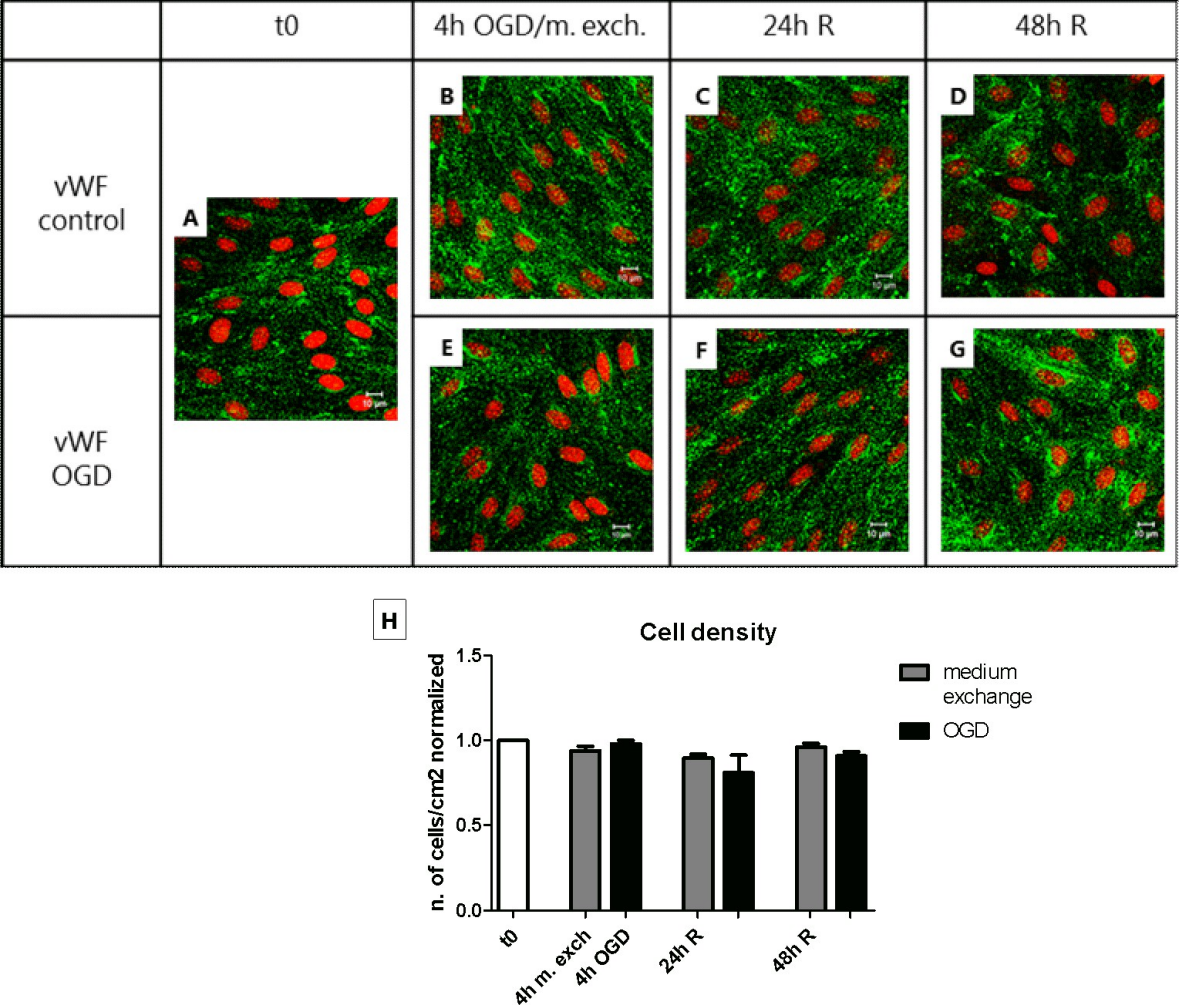
Immunocytochemical evaluation of endothelial cell monolayer purity and determination of the cell density along the oxygen-glucose deprivation (OGD) and medium exchange protocols. Endothelial cell monolayers co-cultured with astrocytes were treated with OGD or medium exchange and reperfused for 24/48 h. (A-G) For each condition, the cells were fixed and stained with an antibody anti-vWF (green) and counterstained with propidium iodide for the nuclei (red). Bars = 10 μm. N = 1; n = 3. (H) For each condition, the cell density expressed as number of cells per cm^2^ was calculated as stated in the “Materials and methods” section. Bar graphs represent means normalized to t0 and error bars are +SEM. N = 5, n = 3–4. The white bar shows the value at t0, the gray bars show the cells subjected to medium exchange, while the black bars show the OGD treated cells. Columns were compared to t0 using one-way ANOVA and Dunnett’s multiple comparison post-test. Bonferroni’s post-test was utilized to compare each pair of columns.

**Fig 4:**
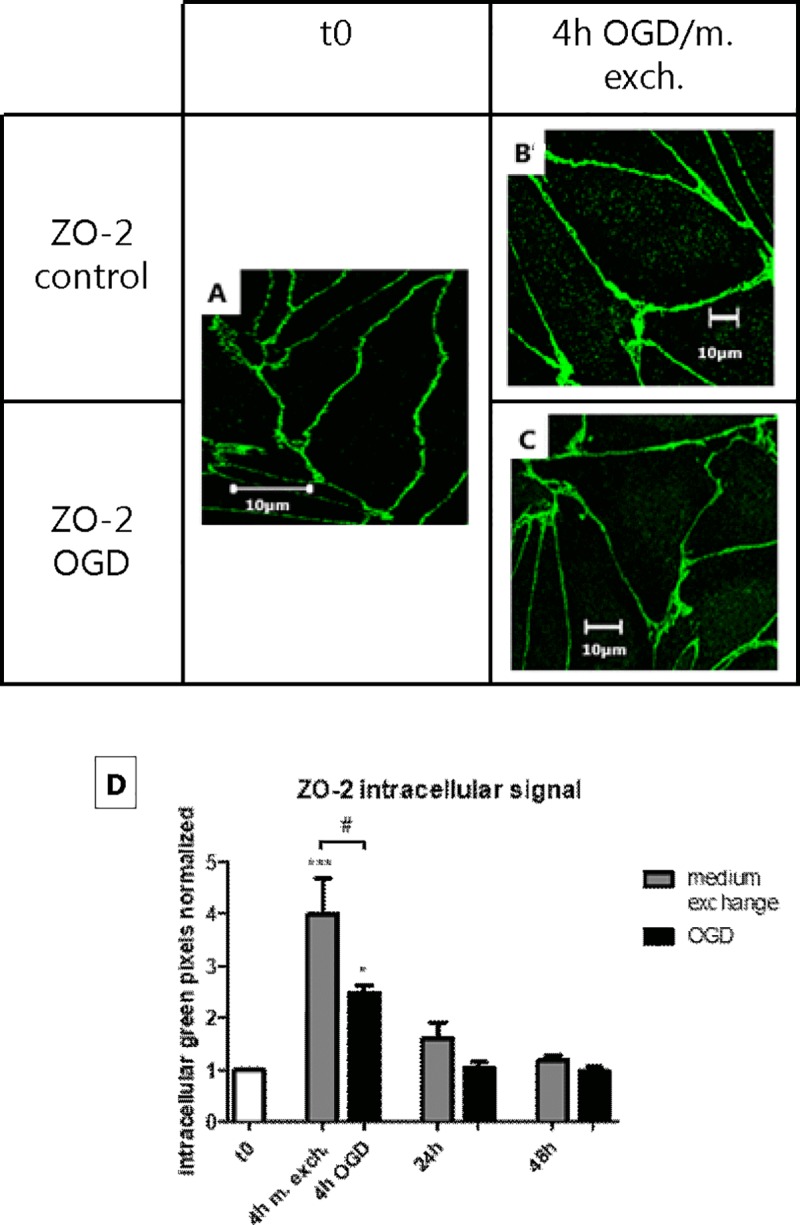
Cellular localization of ZO-2 along the oxygen-glucose deprivation (OGD) and medium exchange protocols. Endothelial cells in co-culture with astrocytes treated with OGD or medium exchange and reperfused for 24/48 h, were fixed and stained with an antibody anti-ZO-2. Fig A shows the localization of ZO-2 before the treatments (t0), Fig B shows the localization of ZO-2 after 4 h from medium exchange, and Fig C shows the localization of ZO-2, 4 h after OGD. Bars = 10 μm. N = 1; n = 3. (D) For each condition, the intracellular signal intensity was estimated using ImageJ as described in the “Materials and methods” section. Bar graphs represent means normalized to t0 and error bars are +SEM. (N = 9–12, n = 3–4). The white bar shows the value at t0, the gray bars show the cells subjected to medium exchange, while the black bars show the OGD treated cells. Columns were compared to t0 using one-way ANOVA and Dunnett’s multiple comparison post-test. *: p<0.05, ***: p<0.001. Bonferroni’s post-test was utilized to compare each pair of columns. #: p<0.05.

**Fig 5:**
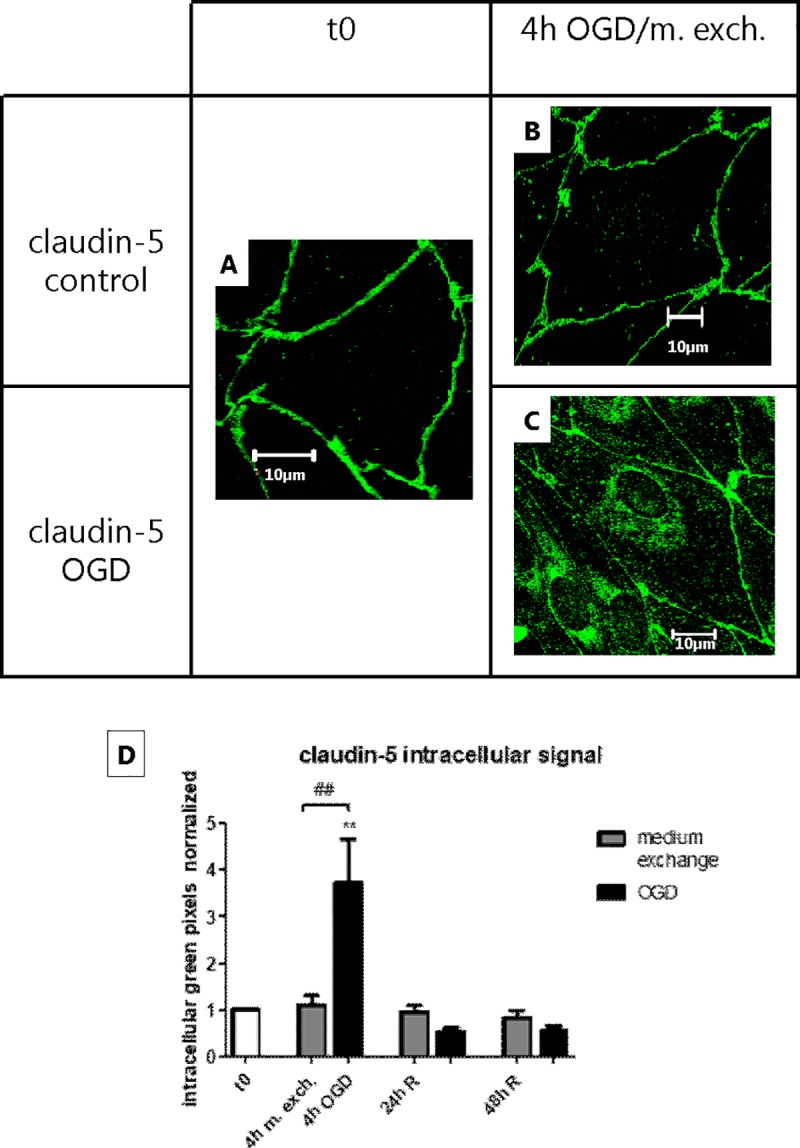
Claudin-5 subcellular localization along the oxygen-glucose deprivation (OGD) and medium exchange. Figs A-C show antibody staining of Claudin-5 under the different treatments. Bars = 10 μm. N = 1; n = 3. (D) For each condition, the intracellular signal intensity was estimated using ImageJ as described in the “Materials and methods” section. Bar graphs represent means normalized to t0 and error bars are +SEM. (N = 9–12, n = 3–4). The white bar shows the value at t0, the gray bars show the cells subjected to medium exchange, while the black bars show the OGD treated cells. Columns were compared to t0 using one-way ANOVA and Dunnett’s multiple comparison post-test. **: p<0.01. Bonferroni’s post-test was utilized to compare each pair of columns. ##: p<0.01.

The endothelial cell monolayers were stained for von Willebrand factor (vWF), a marker protein selectively synthetized and secreted to the blood by the endothelium (Fig 3A–3G). The diffuse punctate staining within the cells confirm that the monolayers utilized in the experimental set up were formed by a pure culture of endothelial cells, without any contamination by other cell types. The cellular localization of claudin-5 and ZO-2 was then analyzed by immunocytochemistry in combination with confocal imaging. As shown in Fig 4, 4 h of OGD treatment led to a visible increase in cytoplasmic ZO-2 signal (approx. 2 folds), which returned to normal after 24/48 h of reperfusion (S1 Fig). However, an even larger increase in ZO-2 intracellular staining was observed in the cells subjected to medium exchange (approx. 4 folds), which also returned to normal after 24 h. Likewise, claudin-5 showed an increase in cytoplasmic signal (approx. 3.5 folds) after 4 h of OGD, which returned to normal in the reperfusion phase. Differently from ZO-2, claudin-5 localization was not affected in the medium exchange-treated cells, indicating a direct effect of the OGD treatment on claudin-5 localization (Fig 5 and S2 Fig).

It was also evaluated whether the number of endothelial cells changed during the OGD/medium exchange protocol. Although no breaches were observed in the monolayers, variation in cell density due to the detachment of the cells could in theory explain the decrease in tightness observed after OGD and medium exchange. However, no significant changes in cell density were observed after 4 h of OGD/medium exchange and after 24/48 h of reperfusion (Fig 3H).

In summary, during the experimental procedure we observed changes in the subcellular localization of TJ proteins. ZO-2 staining significantly increased in the intracellular region in both medium exchange- and OGD-treated monolayers. Claudin-5 only displayed cytosolic localization after the OGD, indicating that claudin-5 was affected specifically by OGD.

### InsR localization was specifically affected by OGD whereas all other receptors and transporters were equally affected by OGD and medium exchange

The mRNA levels of a range of membrane transporters and receptors were investigated in the OGD- and medium exchange-treated brain endothelial cultures. Endogenous transport systems expressed on brain capillary endothelium, such as solute carriers and receptors represent a potential platform for delivering therapeutic molecules to the brain. Changes in their expression and/or localization are therefore of interest for the design of drugs directed toward the ischemic brain tissue.

**Fig 6:**
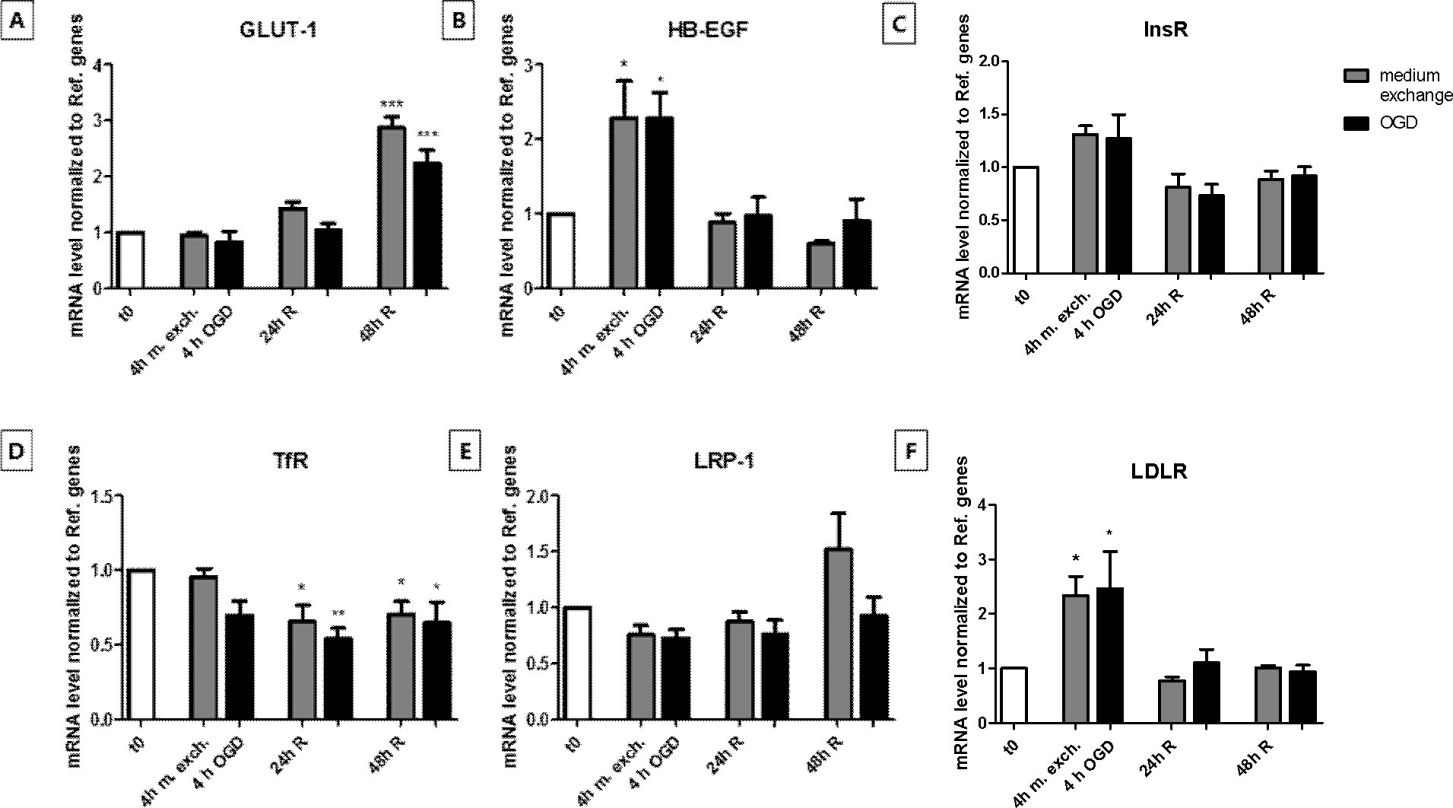
Transcript levels of membrane transporter GLUT-1 and the receptor HB-EGF, InsR, TfR, LDLR and LRP-1 in endothelial cell monolayers before, during and after oxygen-glucose deprivation (OGD) treatment or medium exchange protocol. The transcript level of GLUT-1 (A) and different receptor proteins (B-F) in endothelial cells that had undergone OGD (black bars) or medium exchange (gray bars) were evaluated by RT-qPCR and normalized to reference genes. Primers specifically designed for bovine orthologues were utilized. Bar graphs represent means and error bars are +SEM. N = 3, n = 3–4. Columns were compared to t0 using one-way ANOVA and Dunnett’s multiple comparison post-test. *: p<0.05, **: p<0.01, ***: p<0.001. Bonferroni’s post-test was utilized to compare each pair of columns.

The investigated transcripts were glucose transporter 1 (GLUT-1), the major glucose transporter in the mammalian BBB, heparin-binding EGF-like growth factor (HB-EGF), a growth factor that in its pro-form anchored to the cell membrane has been shown to act as receptor for diphtheria toxin, insulin receptor (InsR), transferrin receptor (TfR), low density lipoprotein receptor (LDLR) and low density lipoprotein receptor-related protein 1 (LRP-1). In our experiments, GLUT-1 mRNA level was significantly upregulated by 48 h of reperfusion following both OGD and medium exchange (Fig 6A). HB-EGF and LDLR transcript levels were upregulated after both 4 h of OGD and medium exchange (Fig 6B and 6F), TfR level did not undergo significant changes after 4 h of OGD and medium exchange, but it decreased in the reperfusion phase (Fig 6D), while InsR and LRP-1 mRNA levels did not display any significant change during the OGD or medium exchange experimental protocols (Fig 6C and 6E).

**Fig 7:**
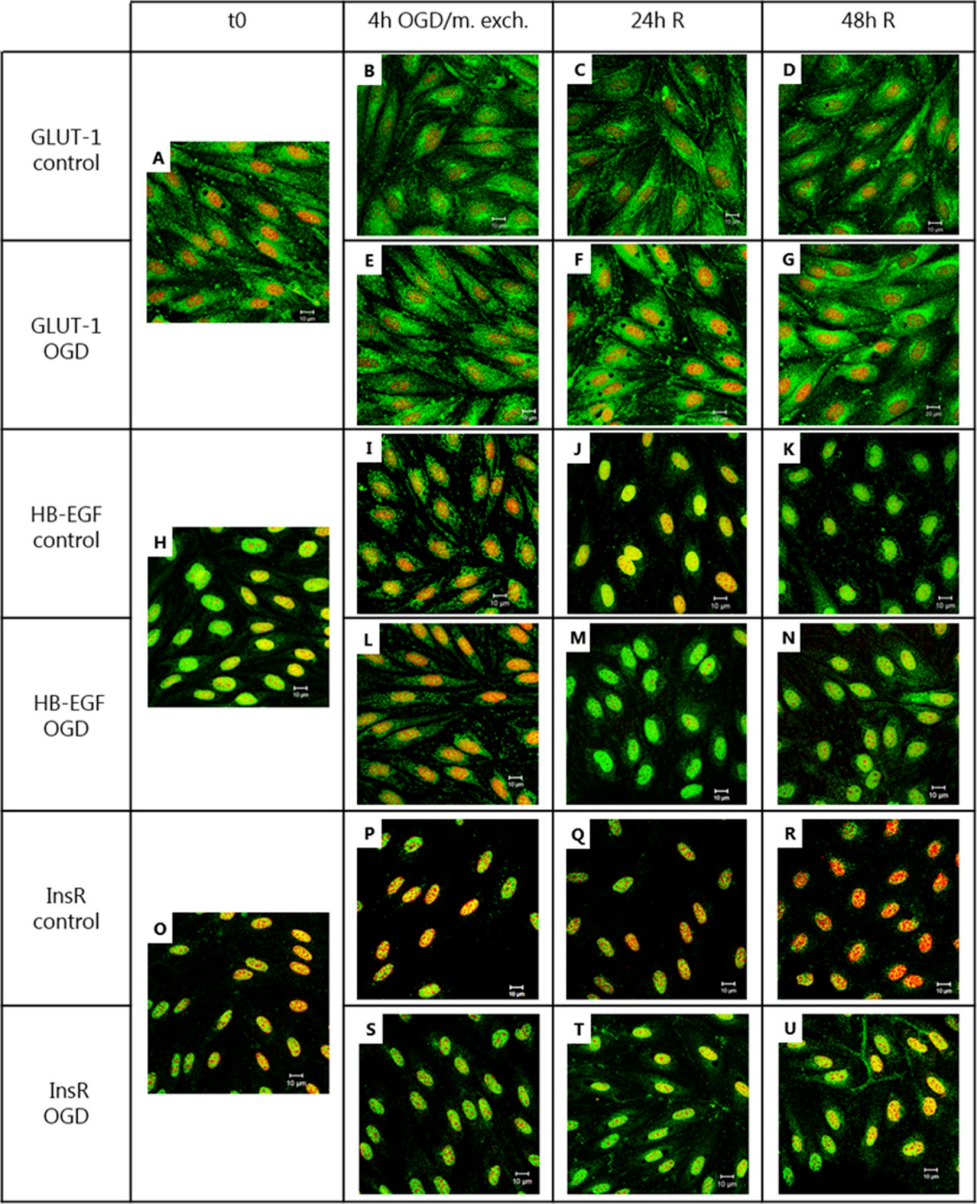
Immunocytochemistry analysis of GLUT-1, HB-EGF and InsR protein localization before, during and after oxygen-glucose deprivation (OGD) and medium exchange treatment. Endothelial cells co-cultured with astrocytes were subjected to OGD for 4 h or to medium exchange, followed by 24/48 h of reperfusion. For each condition, a filter insert was collected, fixed and stained with an antibody against GLUT-1 (A-G), HB-EGF (H-N) or InsR (O-U) (green), and counterstained with propidium iodide for visualizing the cell nuclei (red). Bars = 10 μm. N = 1, n = 3.

As judged by immunolabelling and confocal laser microscopy analysis, GLUT-1 appeared to be localized mainly in the cytosol at t0, after 4 h of OGD and medium exchange and at 48 h of reperfusion following both the OGD and the medium exchange treatments. At 24 h of reperfusion, a slight up-concentration at the cell border was detected in both the OGD- and medium exchange-treated cells (Fig 7A–7G). The receptor HB-EGF had a perinuclear staining with a marked co-localization with the area of the nucleus at t0, 24 h of reperfusion and 48 h of reperfusion upon both treatment protocols, while after 4 h of OGD and medium exchange, the localization appeared to be more diffuse in the cytosol (Fig 7H–7N). InsR seemed to co-localize with the area of the nucleus at t0, 4 h of OGD and medium exchange. At 24 and 48 h of reperfusion after OGD, a slight up-concentration of the signal was detected at the cell border. This phenomenon was not observed in cells that had undergone medium exchange (Fig 7O–7U).

Overall, the OGD- and medium exchange-treated cells showed marked but similar changes in the mRNA levels of GLUT-1, HB-EGF, TfR and LDLR. These changes are thus not likely to be related to the OGD treatment. However, the InsR localization seemed to be specifically affected by the OGD treatment.

### OGD but not medium exchange influenced MRP-1 and P-gp mRNA level and localization

**Fig 8:**
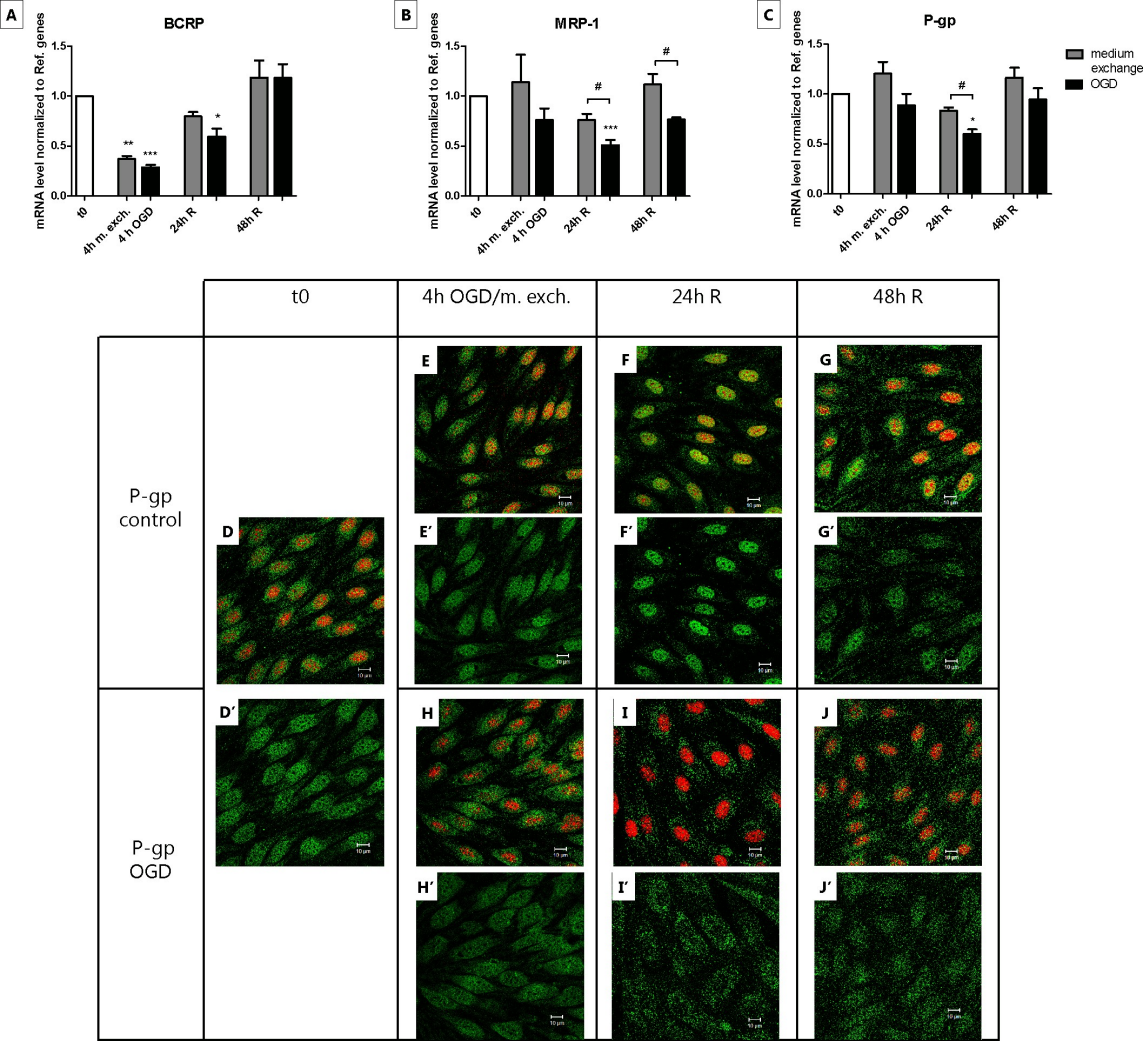
Transcript level of BCRP, MRP-1 and P-gp and cell localization of P-gp in endothelial cells before, during and after 4 h of oxygen-glucose deprivation (OGD) or medium exchange. The mRNA levels of BCRP (A), MRP-1 (B) and P-gp (C) at the different time points of the OGD (black bars) and medium exchange (gray bars) protocol were measured by RT-qPCR and normalized to reference genes. Primers specifically designed for bovine orthologues were utilized. Bar graphs represent means and error bars are +SEM. N = 3, n = 3–4. Columns were compared to t0 using one-way ANOVA and Dunnett’s multiple comparison post-test. *: p<0.05, **: p<0.01, ***: p<0.001. Bonferroni’s post-test was utilized to compare each pair of columns. #: p<0.05. Figs D-J show antibody staining of P-gp (green), and cell nuclei staining with propidium iodide (red) under the different treatments. Figs D’-J’ show exclusively the P-gp staining from Figs D-J. Bars = 10 μm. N = 1; n = 3.

The brain endothelial cells express a panel of different ABC proteins, including breast cancer resistance protein (BCRP), multidrug resistance-associated protein 1 (MRP1) and P-glycoprotein (P-gp). Changes in their expression level and/or subcellular localization can influence the possibility for a drug to be delivered into the brain, and variations have been reported during ischemic stroke [21, 22]. We therefore evaluated the transcript level and localization of selected ABC transporters in the OGD treated cells and compared the results with the cells subjected only to medium exchange. The analysis of the mRNA level of BCRP after 4 h of OGD revealed a downregulation with a total recovery after 48 h of reperfusion. In the cells subjected to medium exchange, the transcript level of BCRP was subjected to a similar drop after 4 h from medium exchange, but the recovery seemed to be faster since the normal level was reached already after 24 h of recovery (Fig 8A). The MRP-1 and P-gp mRNA levels displayed a significant downregulation after 24 h of reperfusion in the OGD treated cells, and their initial transcript levels were restored the subsequent day (48 h of reperfusion). These changes were only observed in the OGD treated cells, whereas medium exchange-treated cells did not show any significant changes (Fig 8B and 8C). Confocal imaging of P-gp showed a diffuse expression throughout the cells with some co-localization with the area of the nucleus at t0. Similar staining was observed at each time point for the medium exchange-treated cells, while the OGD treated cells after 24 h from the treatment showed a staining predominantly in the cytosol (Fig 8D–8J). In summary, no significant differences were found in the BCRP mRNA amount when applying the two different experimental protocols, while the transcript levels of MRP-1 and P-gp decreased after 24 h of reperfusion only in the OGD treated cells. Furthermore, the decrease of P-gp mRNA level at 24 h of reperfusion was accompanied with a change in protein staining observed only in the OGD treated cells.

## Discussion

The use of *in vitro* models of the BBB to investigate the molecular mechanisms underlying drug permeation or specific pathologies is attractive, since experimental conditions can be strictly controlled, and the tissue is easily accessible for studies of mRNA and protein expression, as well as for studies of signaling transport pathways.

Various *in vitro* models have been used to mimic the ischemic BBB. They differ regarding the type of cells used (primary, immortalized or differentiated from stem cells), the cell origin (human, mouse, rat bovine, etc.), the cells included in the model (endothelial cells, astrocytes, pericytes, neurons) and the parameters selected for the treatment (levels of oxygen and glucose and duration of the treatment) [23].

The most common experimental protocol used to model ischemic stroke *in vitro* is OGD. This requires the incubation of the cells in an environment with a low oxygen content and the change of the culture medium to a medium with a low or no glucose. To mimic *in vivo* ischemic stroke [24, 25], we treated our endothelial cells with 0 mM of glucose and 1% of O_2_ for 4 h, follow by a recovery period (“reperfusion”) for 48 h.

The brain capillary endothelial cells are cells very thin (from 0.7±0.2 μm at the cell edges to 2.4±1.3 μm at the perinuclear region) [10] and fragile and express several mechanosensors which enable the translation of mechanical stimuli into molecular responses. The change of the medium occurring during OGD might potentially be interpreted as mechanical stress and compromise the endothelial monolayer integrity. We therefore included controls, where we replaced the culture medium with fresh medium and incubated cultures under standard conditions (90% room air-10% CO_2_, 37°C). 4 h of OGD and simple medium exchange induced similar changes in endothelial monolayer permeability, junction protein transcript levels and ZO-2 subcellular localization, whereas the localization of claudin-5 was specifically affected by OGD. These results suggested that the changes observed under both OGD and in medium exchange controls, were due to the mechanical stress caused by the change of the medium, but also that the alterations in claudin-5 localization were specifically triggered by OGD. A similar pattern of claudin-5 localization and transcript level alteration was described by Koto T. et al. [26]. bEND.3 cells treated with 24 h of hypoxia displayed a significant decrease in claudin-5 protein level and a reduced relocation of this protein at the plasma membrane, which coincided with weaker barrier properties and lower TEER. Neuhaus W. et al. [25] showed that claudin-5 gene expression level was decreased after OGD and that its presence at the plasma membrane was altered by this treatment.

Another specific change induced by the OGD was the marked increase in membrane in localization of InsR in the reperfusion phase after OGD (but not after medium exchange). InsR mediates insulin signaling, uptake and degradation [27]. It has been reported that insulin has several effect on barrier function such as the enhancement of tyrosine and tryptophan transport, the increase of the blood-to-brain transport of leptin, the increase of the expression and function of the efflux transporter P-gp, and the decrease of BCRP [28]. On the other hand, insulin does not seem to be involved in the maintenance of TJ integrity [29].

In our model, the mRNA level of P-gp and MRP-1 slightly diminished after 24 h of reperfusion following OGD but not after medium exchange. In the same way, the change in localization of P-gp was observed only after OGD and not in the medium exchange protocol. Regarding P-gp, these results are in contrast with what was found *in vivo* by Spudich et al., where mice subjected to intraluminal middle cerebral artery occlusion (MCAO) displayed an enhanced expression and activity of P-gp in the capillary endothelium [30]. On the other hand, no significant differences in P-gp mRNA amount were found between the periinfarct and the contralateral region in rats undergone MCAO treatment [21]. Previous studies on brain endothelial cells in culture also showed conflicting results. Patak P. et al. using an *in vitro* model of BBB based on hCMEC/D3 cell line did not find any significant change in P-gp protein and mRNA expression level after 48 h of hypoxia [31]. It has to be noted that important factors of an ischemic event such as glucose deprivation, reoxygenation and the astrocyte effect were not considered in this study. 6 h of hypoxic treatment followed by 24 h of reoxygenation led to an increased expression of P-gp at both time points in a primary rat brain endothelial cell model [32]. Conversely, Robertson et al. observed a significant increase in P-gp expression only after 24 h of reoxygenation following 6 h of hypoxia in primary and immortalized rat brain endothelial cell model of hypoxia/reoxygenation [33]. A more recent study by Neuhaus W. et al. reported that OGD did not produce variations in P-gp transcript level in the immortalized mouse cell line cerebEND cultured with astrocytes-derived medium, as compared to normoxic endothelial cell cultured in the same condition, while endothelial cells without astrocytes-derived medium responded to OGD with a significant upregulation of P-gp gene expression [25]. Corresponding results were obtained also at protein level.

The data reported in literature about P-gp expression upon OGD/MCAO treatments appear clearly controversial. A downregulation of P-gp expression would however be of great interest in terms of drug delivery, because it would allow the delivery of drugs, which under physiological conditions would be pumped out from the cell. Bi-directional transport studies using a P-gp substrate in combination with P-gp inhibitors would help to elucidate whether the changes observed at mRNA level can be translated into functional differences.

Finally, to our knowledge, not much is known about MRP-1 expression in ischemic conditions. A study by Kilic E. et al. described that the expression level of MRP-1 slightly decreased in mice subjected to 3 h of MCAO [34]. This was in line with what we found in our *in vitro* model.

In the present study, we clearly demonstrated that most of the changes observed after OGD and reperfusion were due to the exchange of the medium and were not direct effects of the OGD treatment. Under physiological conditions, endothelial cells are continuously exposed to a fluid shear stress exercised by the blood flow. It has been observed that a laminar shear stress produces an enhancement of barrier properties in cultured endothelial cells. This seems to be mediated by mechanosensitive complexes which are constitutively present in endothelial cells and are capable to transduce the shear stress stimuli in an intracellular regulatory signal [6–8, 35]. Conversely, a disturbed flow [36] or an augmented shear stress [37] cause an increase in endothelial monolayer permeability. These findings suggest that the change of culture medium, which represent a non-physiological liquid movement in the proximity of endothelial cells, may interfere with different pathways involved in barrier regulation and in particular, it seems to be a potent stimulus of endothelial cell transcript level changes. The alterations in transcript levels that in earlier *in vitro* studies not using medium exchange controls have been attributed to ischemic stress, might therefore be ascribed to medium exchange in itself rather than to OGD.

### Conclusions and perspectives

In the present study, we demonstrated that OGD specifically causes alteration in claudin-5, InsR and P-gp localization and in MRP-1 and P-gp mRNA levels in co-cultured brain endothelial cells. However, simple medium exchange perturbs functional barrier properties to the same degree as observed after OGD. Furthermore, both medium exchange and OGD induce changes in the transcript levels for a number of TJ and TJ-associated proteins, as well as a number of other endothelial targets. This makes it difficult to detect specific effects of OGD on vectorial transport parameters. Flow systems, where the endothelial monolayers are continuously perfused by circulating culture medium whose composition can be manipulated might offer better conditions to investigate the effects of OGD *in vitro*.

## Supporting information

S1 Table Overview of the culture media and buffers used for cell culture and for the experiment procedures. FBS = Fetal Bovine Serum, cAMP = cyclic Adenosine monophosphate, TES = N-Tris(hydroximethil)metil-2-aminoethanesulfonoc Acid.
(TIF)

S2 Table Overview of the applied primers. (TIF)

S3 Table Overview of the employed antibodies. (TIF)

S1 Fig Cellular localization of ZO-2 along the OGD and medium exchange protocols. Endothelial cells in co-culture with astrocytes treated with OGD or medium exchange and reperfused for 24/48 h, were fixed and stained with an antibody anti-ZO-2 (green). The cell nuclei were visualized with propidium iodide staining (red). Fig A shows the localization of ZO-2 before the treatments (t0). Figs B-D show the localization of ZO-2 after 4 h from medium exchange, after 24 h of reperfusion following medium exchange and after 48 h of reperfusion following medium exchange, respectively. Figs E-G show the localization of ZO-2 after 4 h of OGD, after 24 h of reperfusion following OGD and after 48 h of reperfusion following OGD, respectively. Figs A’-G’ show solely the distribution of ZO-2 (green signal) in a single cell magnified from Figs A-G respectively. Bars = 10 μm. N = 1; n = 3. (H) For each condition, the intracellular green signal intensity was estimated using ImageJ as described in the “Materials and methods” section. Bar graphs represent means normalized to t0 and error bars are +SEM. (N = 9–12, n = 3–4). The white bar shows the value at t0, the gray bars show the cells subjected to medium exchange, while the black bars show the OGD treated cells. Columns were compared to t0 using one-way ANOVA and Dunnett’s multiple comparison post-test. *: p<0.05, ***: p<0.001. Bonferroni’s post-test was utilized to compare each pair of columns. #: p<0.05.
(TIF)

S2 Fig Claudin-5 subcellular localization along the OGD and medium exchange. Figs A-G show antibody staining of Claudin-5 (green), and cell nuclei staining with propidium iodide (red) under the different treatments. Fig A’- G’ shows exclusively the Claudin-5 staining from Figs A-G. Bars = 10 μm. N = 1; n = 3. (H) For each condition, the intracellular green signal intensity was estimated using ImageJ as described in the “Materials and methods” section. Bar graphs represent means normalized to t0 and error bars are +SEM. (N = 9–12, n = 3–4). The white bar shows the value at t0, the gray bars show the cells subjected to medium exchange, while the black bars show the OGD treated cells. Columns were compared to t0 using one-way ANOVA and Dunnett’s multiple comparison post-test. **: p<0.01. Bonferroni’s post-test was utilized to compare each pair of columns. ##: p<0.01.
(TIF)
