# Supplementary figures and images for: Effects of oxygen-glucose deprivation (OGD) on barrier properties and mRNA transcript levels of selected marker proteins in brain endothelial cells/astrocyte co-cultures
Source: PLoS One. 2019 Aug 19;14(8):e0221103. doi: 10.1371/journal.pone.0221103 (PMC6699694; doi:10.1371/journal.pone.0221103)

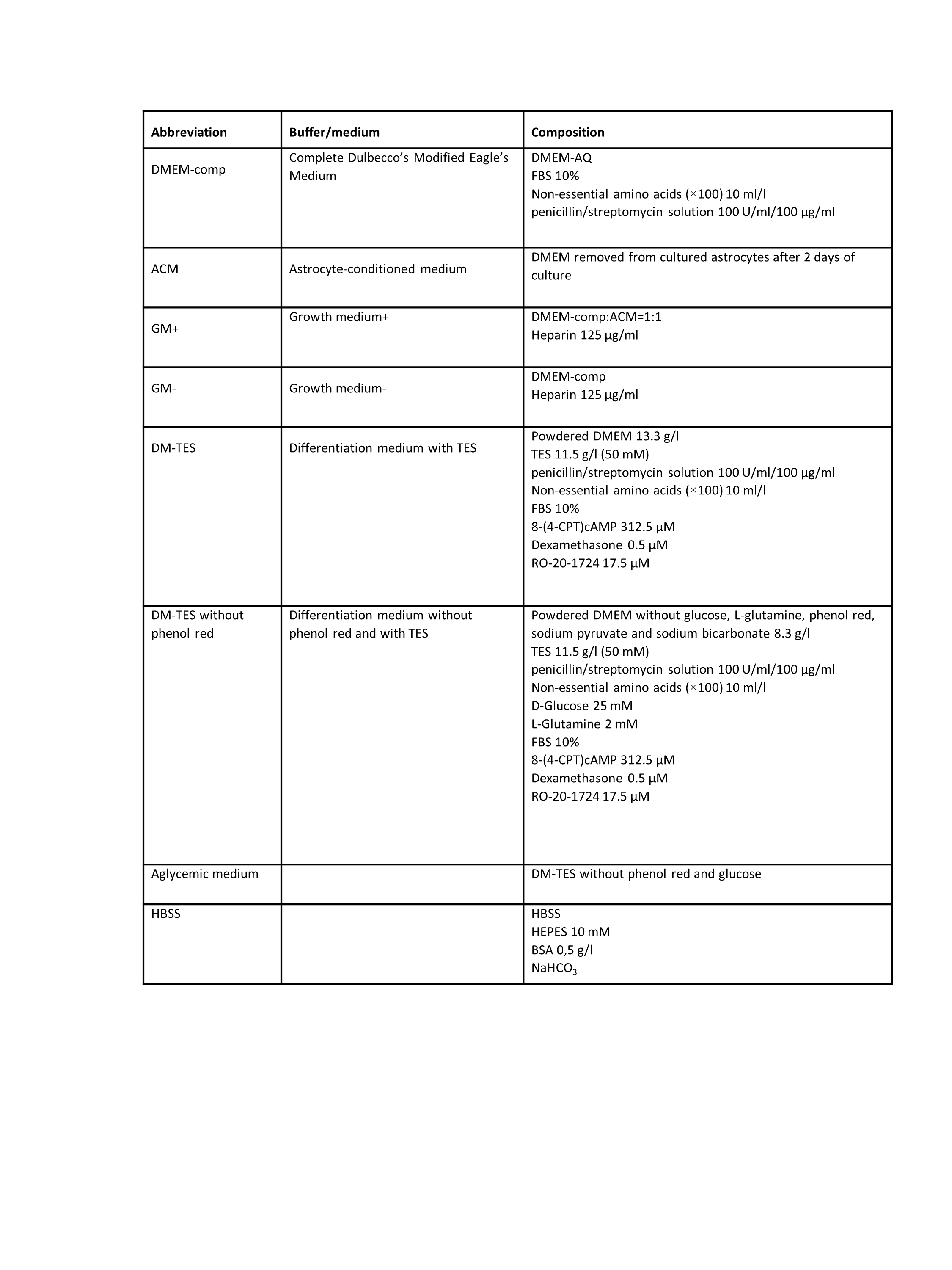

Supplement: S1 Table — FBS = Fetal Bovine Serum, cAMP = cyclic Adenosine monophosphate, TES = N-Tris(hydroximethil)metil-2-aminoethanesulfonoc Acid. (TIF) [file pone.0221103.s001.tif]

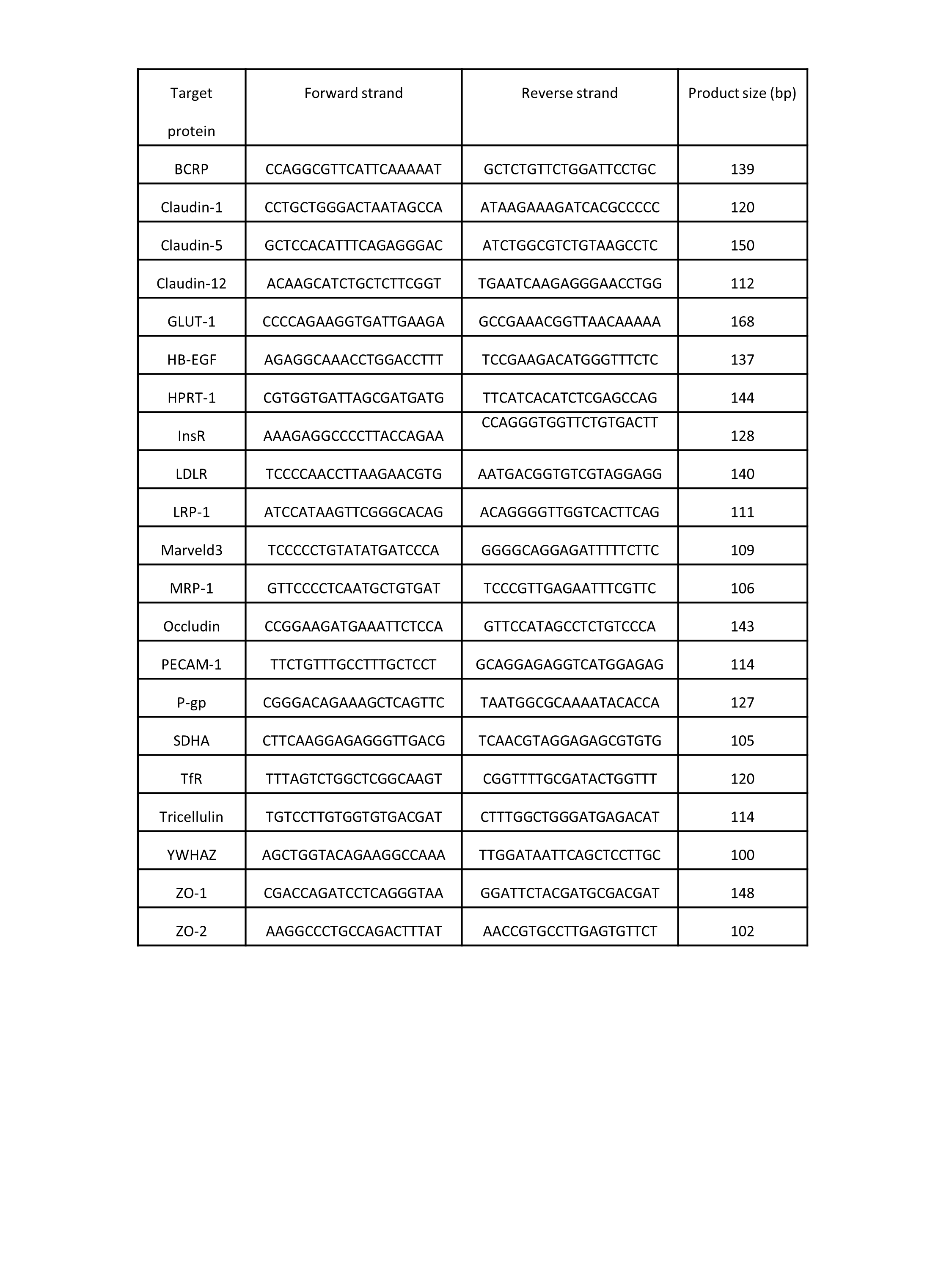

Supplement: S2 Table — (TIF) [file pone.0221103.s002.tif]

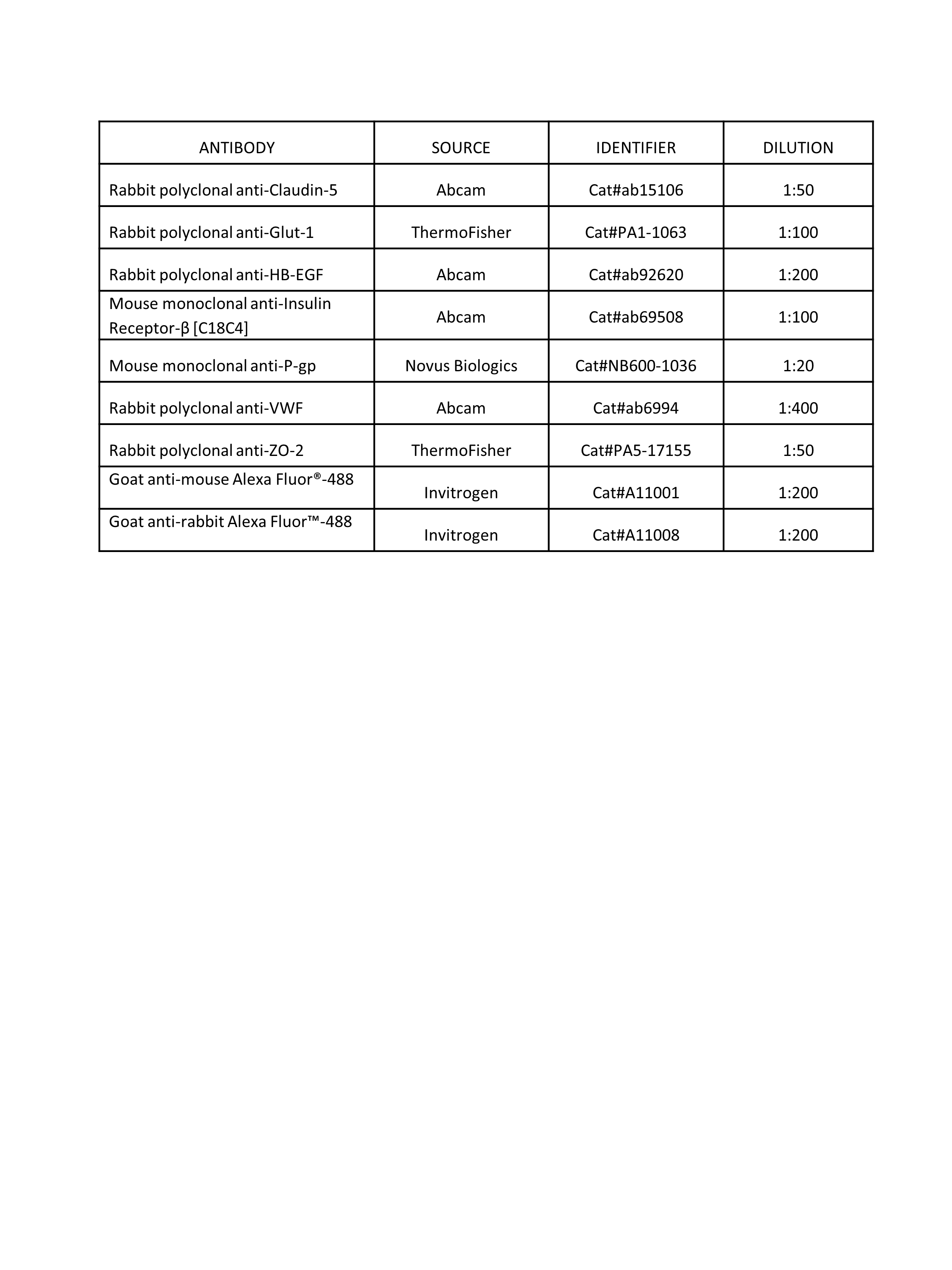

Supplement: S3 Table — (TIF) [file pone.0221103.s003.tif]

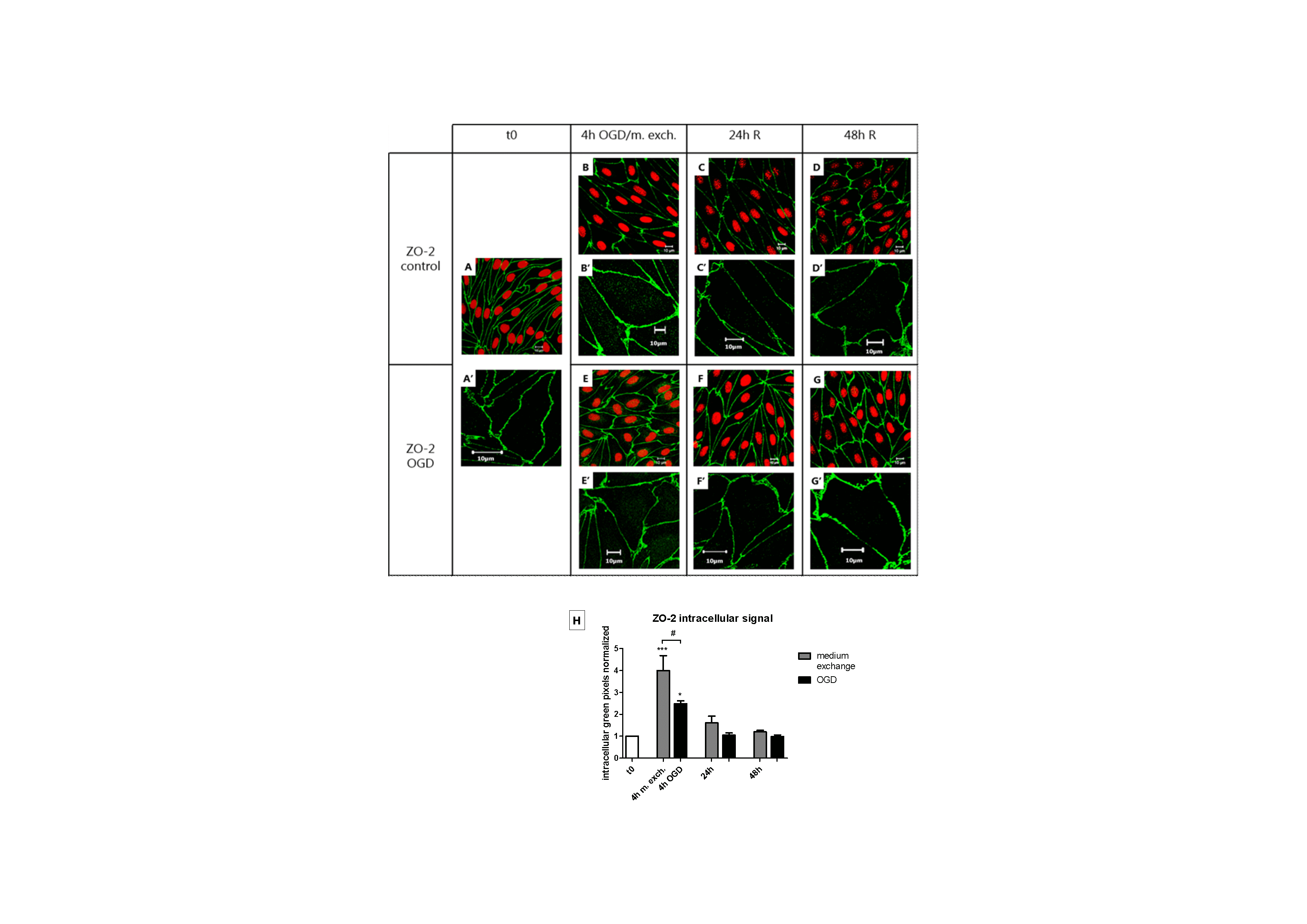

Supplement: S1 Fig — Endothelial cells in co-culture with astrocytes treated with OGD or medium exchange and reperfused for 24/48 h, were fixed and stained with an antibody anti-ZO-2 (green). The cell nuclei were visualized with propidium iodide staining (red). Fig A shows the localization of ZO-2 before the treatments (t0). Figs B-D show the localization of ZO-2 after 4 h from medium exchange, after 24 h of reperfusion following medium exchange and after 48 h of reperfusion following medium exchange, respectively. Figs E-G show the localization of ZO-2 after 4 h of OGD, after 24 h of reperfusion following OGD and after 48 h of reperfusion following OGD, respectively. Figs A’-G’ show solely the distribution of ZO-2 (green signal) in a single cell magnified from Figs A-G respectively. Bars = 10 μm. N = 1; n = 3. (H) For each condition, the intracellular green signal intensity was estimated using ImageJ as described in the “Materials and methods” section. Bar graphs represent means normalized to t0 and error bars are +SEM. (N = 9–12, n = 3–4). The white bar shows the value at t0, the gray bars show the cells subjected to medium exchange, while the black bars show the OGD treated cells. Columns were compared to t0 using one-way ANOVA and Dunnett’s multiple comparison post-test. *: p<0.05, ***: p<0.001. Bonferroni’s post-test was utilized to compare each pair of columns. #: p<0.05. (TIF) [file pone.0221103.s004.tif]

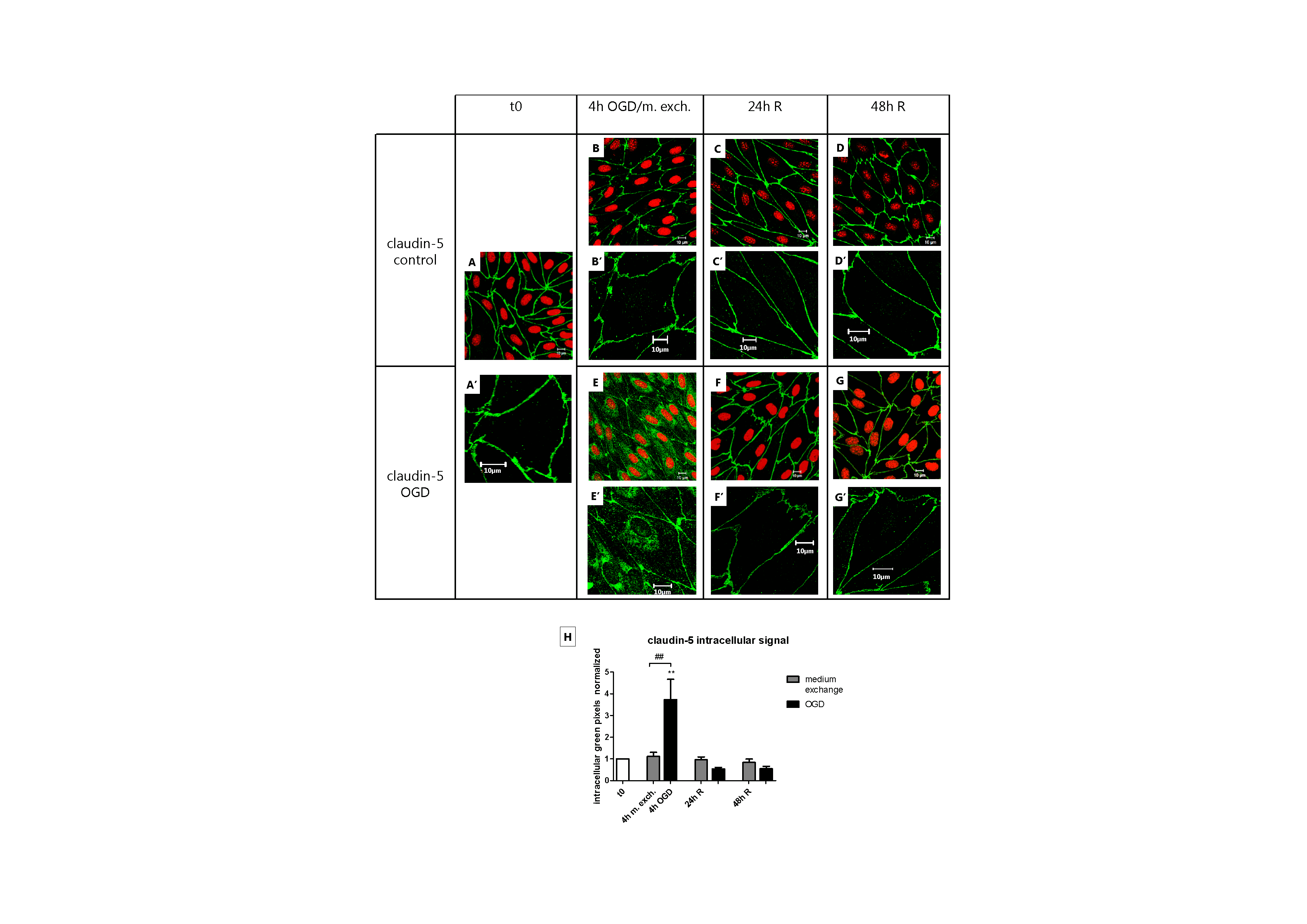

Supplement: S2 Fig — Figs A-G show antibody staining of Claudin-5 (green), and cell nuclei staining with propidium iodide (red) under the different treatments. Fig A’- G’ shows exclusively the Claudin-5 staining from Figs A-G. Bars = 10 μm. N = 1; n = 3. (H) For each condition, the intracellular green signal intensity was estimated using ImageJ as described in the “Materials and methods” section. Bar graphs represent means normalized to t0 and error bars are +SEM. (N = 9–12, n = 3–4). The white bar shows the value at t0, the gray bars show the cells subjected to medium exchange, while the black bars show the OGD treated cells. Columns were compared to t0 using one-way ANOVA and Dunnett’s multiple comparison post-test. **: p<0.01. Bonferroni’s post-test was utilized to compare each pair of columns. ##: p<0.01. (TIF) [file pone.0221103.s005.tif]
